# Effect of high-intensity childhood games on heart rate variability, saliva leptin concentrations, and body composition in children

**DOI:** 10.1590/1414-431X2025e14479

**Published:** 2025-06-16

**Authors:** C.Y. Rodriguez-Triviño, S. Quintana, C.E. Osorio-Vélez, M. Garcia-Florez

**Affiliations:** 1 School of Nursing, Faculty of Health, Universidad del Valle, Santiago de Cali, Valle del Cauca, Colombia; 2 Department of Physiology, Medical Faculty, Universidad Nacional de Colombia, Bogotá, Colombia; 3 Carlos Osorio Velez Medical Unit, Neiva, Colombia; 4 Colombian Sports Medicine Association (AMEDCO), Colombia; 5 Faculty of Health Sciences, Universidad Surcolombiana, Neiva, Colombia

**Keywords:** Exercise, Games, Recreational, Body composition, Child, Heart rate variability

## Abstract

The aim of this study was to evaluate the effect of high-intensity childhood games on cardiac autonomic regulation, obesity biomarkers, and body composition in overweight or obese children compared to moderate-intensity games. A single-blind, randomized controlled study was conducted including children aged 6 to 9 years with overweight or obesity. Participants were randomly assigned to two groups: 33 in the moderate-intensity interval game group (MIIG) and 29 in the high-intensity interval game group (HIIG). The intervention lasted 16 weeks, with measurements conducted under double blinding. The study followed institutional ethical standards and was registered on ClinicalTrials.gov (CT NCT05294601). A total of 74 children were recruited, with 7 excluded after sports medicine assessment, leaving 67 children randomized. Five participants dropped out during the study. HRV analysis revealed significant differences in frequency dominance in the HIIG group. High-frequency power, linked to parasympathetic dominance, increased from 59.3 to 65.8 nu (P=0.03), while low-frequency power, related to sympathetic activity, decreased from 40.6 to 34.13 nu (P=0.04). Salivary leptin concentrations decreased significantly from 0.33 to 0.32 ng/mL (P=0.008) in the MIIG group and from 0.35 to 0.32 ng/mL (P=0.004) in the HIIG group. Childhood games positively impacted anthropometry, HRV, and leptin concentrations in both intensity groups, indicating metabolic improvement. However, only the high-intensity strategy enhanced parasympathetic dominance and sympathetic-parasympathetic balance, potentially reducing long-term cardiovascular risk.

## Introduction

Childhood obesity, defined as a body mass index (BMI) above the 85th percentile and/or a high age-related percentage of body fat, is a multifactorial condition with serious health consequences, including early cardiovascular disease (CVD), endocrine dysfunction, and generalized low-grade inflammation (1). Overweight or obese children are much more likely to become obese adults compared to their non-overweight counterparts (1). They may also experience autonomic dysfunction, changes in the vascular system, hearing alterations, and blood pressure control impairment. Skinner et al. (2) reported elevated high-density lipoprotein (HDL) cholesterol, triglycerides, glycosylated hemoglobin, and systolic blood pressure as obesity progressed from class I to III, with a higher prevalence in children and young adults. It has also been determined that obesity, both in children and adults, induces a state of low-grade inflammation and metabolic alterations, such as increased plasma leptin, hyperinsulinemia, and elevated production of tumor necrosis factor-alpha (TNF-α) and interleukin-6 (IL-6), among others (3). Whether autonomic dysfunction or low-grade inflammation in children can be reversed remains unclear. Mazurak et al. (4) found a decrease of parasympathetic dominance in obese children, and Javorka et al. (5) detected subtle changes related to autonomic dysfunction in young obese subjects using nonlinear analysis and multiscale entropy analysis. Reduced heart rate variability (HRV) has been associated with an increased risk of CVD, as it has been observed in individuals with hypertension, diabetes, hypercholesterolemia, and unhealthy lifestyle habits such as physical inactivity and tobacco use (6).

Regular physical exercise plays a crucial role in maintaining a healthy weight in both adults and children, contributing to improved autonomic control and cardiovascular health. (7). High-intensity interval training (HIIT) has emerged as a complementary approach in managing childhood obesity by promoting metabolic and cardiovascular improvements (7). Dias et al. (8) suggest that HIIT can provide similar or superior health benefits compared to moderate or low-intensity exercise, but in a shorter time frame. Despite its potential, barriers remain to its implementation among school-age children, including the need for age-appropriate programs that engage families and educational institutions. Integrating HIIT into childhood games offers a feasible and motivating alternative, as these activities naturally involve alternating periods of intense exercise and rest (9). This playful approach enhances participation while supporting both physical fitness and emotional well-being. Further clinical evidence is needed to standardize protocols and confirm long-term benefits in reducing obesity-related risk factors (8).

In children, evidence regarding the association between autonomic dysfunction and body composition remains contradictory, as does its relationship with salivary concentrations of leptin, adiponectin, insulin, IL-6, and TNF-α (3). It is also unclear whether these variables can be modified through game-based training. Additionally, the inflammatory profile generated by obesity requires research into new minimally invasive markers measured in saliva. Therefore, this study aimed to evaluate the effect of high-intensity childhood games compared to moderate-intensity games on cardiac autonomic regulation, obesity biomarkers, and body composition in boys and girls aged 6 to 9 years with obesity or overweight.

## Material and Methods

A prospective design with a 16-week intervention of high-intensity interval games (HIIG) was compared with regular moderate-intensity interval physical education based on games (MIIG), incorporating double blinding (both subjects and evaluators of pre-and post-tests). All procedures followed institutional ethical standards and were registered on clinicaltrials.gov with the code NCT05294601. The interventions adhered to the Standard Protocol Items: Recommendations for Interventional Trials (SPIRIT) recommendations for experimental studies.

A general weight and height screening was conducted at the school to identify children with some degree of overweight or obesity. Families and children meeting these criteria were invited to participate in the study. The final sample included 67 children randomly assigned to the control group (MIIG) and the experimental group (HIIG), selected from students enrolled in schools that agreed to participate in the study and met the established inclusion criteria: signed informed consent from the legal guardian, voluntary assent from the child, weight above the 85th percentile, Tanner stage below II, and public or private health insurance.

Children with musculoskeletal conditions limiting physical activity, chronic diseases such as chronic asthma, kidney disease, or diabetes, use of medications affecting body composition or insulin secretion such as glucocorticoids, evidence of heart disease or pediatric hypertension defined as blood pressure above the 95th percentile for systolic or diastolic values, diagnosed attention deficit hyperactivity disorder, and those who, at medical discretion, could not participate or showed symptoms such as dyspnea, chest pain, or palpitations, among others, during the physical fitness test were excluded from the study.

Random allocation to the groups was performed after the initial assessments using simple random sampling with the Epidat version 4.2 software, ensuring masking. A total of 30 boys and 32 girls completed the study (Table 1).

**Table 1:** General characteristics of the subjects.

Characteristics	Boys (n=30)	Girls (n=32)
Age (years)	8.2 (0.1)	7.9 (0.1)
Height (cm)	132.0 (1.2)	131.8 (1.1)
Weight (kg)	36.0 (0.3)	37.7 (1.3)
Body mass index percentile	93.4 (0.9)	93.2 (1.0)

Data are reported as mean and SD (n=62).

Parents of children excluded from the study due to specific exclusion criteria, such as the presence of chronic illnesses or findings indicating risk, were informed about these findings, and the children were referred to appropriate medical services according to local clinical recommendations, thus ensuring compliance with ethical considerations supervised by the Ethics Committee that approved the project. Both the children and their parents benefited from educational activities, regardless of their participation in the trial. Additionally, parents' concerns related to the established medical management and the development of school activities were addressed.

**Figure 1:**
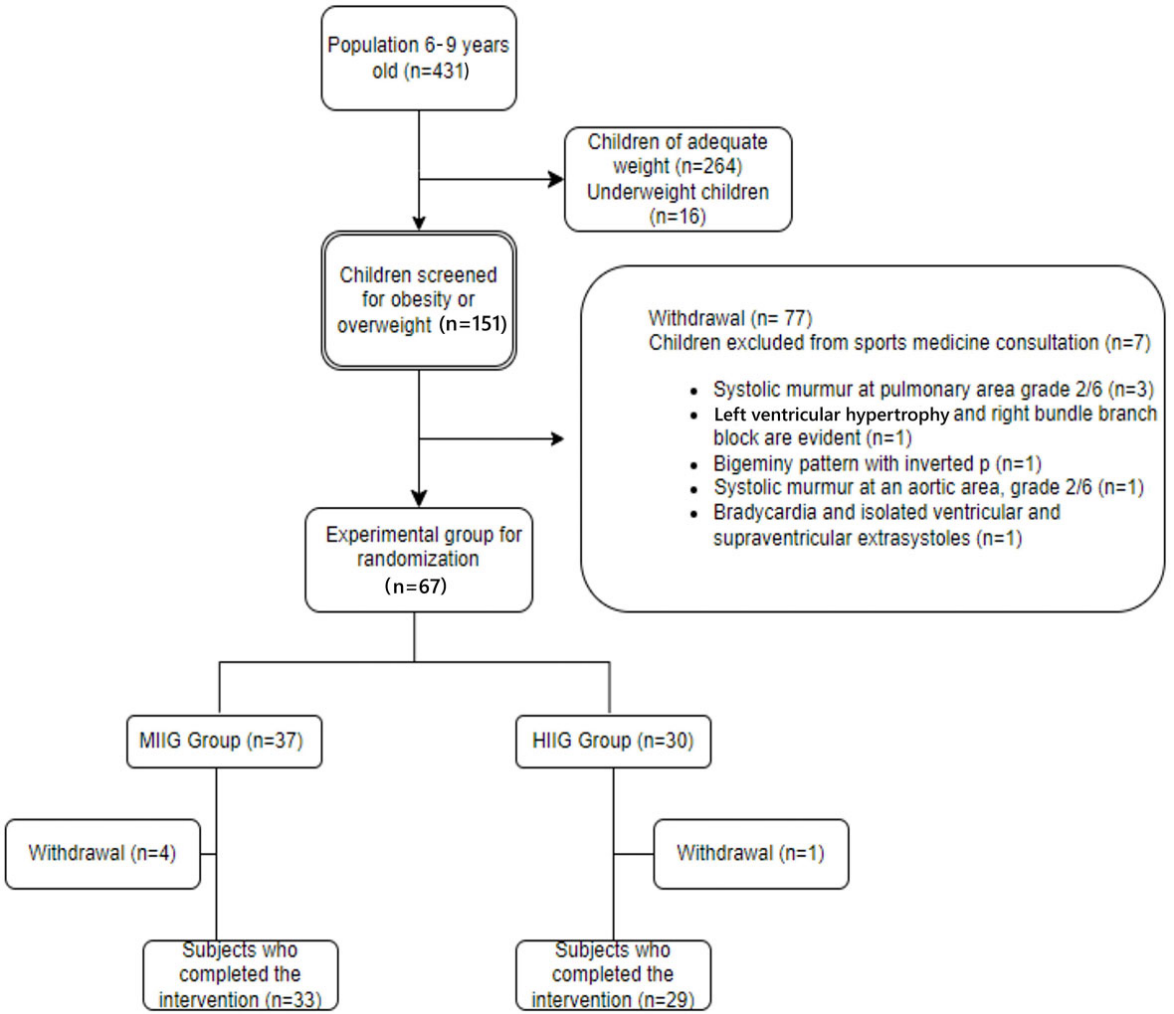
Methodology flowchart. MIIG: moderate-intensity interval game group; HIIG: high-intensity interval game group.

The project unfolded in four phases (Figure 1). Initially (phase I), screening for weight and height was conducted on all 6- to 9-year-old children in the school (a total of 431 children), of whom 31% exhibited some degree of obesity or were overweight. In phase II, children identified as obese or overweight (n=151) were invited along with their guardians to sign the informed consent and assent. Seventy-four at-risk subjects attended. All children underwent assessment by a sports medicine physician, including cardiovascular assessment and examination of cardiac and pulmonary areas. Children with murmurs above 2/6 or with electrocardiographic abnormalities unrelated to age and at the discretion of the physician were referred to pediatrics. The evaluation criteria followed the Pediatrics Association's Clinical Guidelines for Cardiovascular Evaluation Before Sports Practice in Pediatrics. This included a complete physical examination and a 12-lead resting electrocardiogram (ECG) using a Schiller electrocardiograph.

For HRV determination, the PowerLab^®^ hardware, which includes the LabChart^®^ software for data recording, was used. The data were exported to KUBIOS^®^ for analysis in three dimensions: time domain, frequency domain, and nonlinear domain. At least 10 min of lead II ECG were recorded using LabChart^®^, enabling cyclic measurements in the channel settings, selecting the appropriate measurement source and preset as indicated by the manufacturer, and activating the R markers. RR intervals were verified for accuracy, non-numeric rows were deleted, and the file was saved as “Data Pad Only” in text format (txt). Since the file used commas as decimal separators, they were replaced with periods using the Edit/Replace option to ensure compatibility with KUBIOS^®^, where the file was imported and the different HRV dimensions were analyzed.

Before the recording, subjects were in a basal state, in a quiet environment with a temperature of 21 to 24°C and humidity between 40 and 60%, having abstained from caffeinated beverages. Children were instructed to remain at rest and in silence. Seven children with heart murmurs and electrocardiographic changes were excluded and referred to pediatric cardiology (Figure 1).

In phase III, children who passed the blinded cardiovascular assessment underwent a “Course Navette” test to determine baseline maximal oxygen consumption (VO_2_ max), validated for ages 6 to 15 by Leger et al. (10). Additionally, unstimulated whole saliva was collected from all subjects between 9 am and noon. Guardians were instructed to ensure that each individual refrained from eating on the day of collection and drinking or brushing their teeth for at least 60 min before collection. Each subject underwent a rinsing process with water before drooling vertically, allowing saliva to drain into the tube. Between two and five milliliters of whole saliva were obtained from each participant. The test tubes were placed in ice at 8°C and subsequently frozen at -80°C to preserve the saliva samples. An enzyme-linked immunosorbent assay (ELISA-sandwich) method was employed to analyze the saliva samples using commercially available immunoassay kits. This analysis aimed to determine the concentrations of IL-6, TNF-α, leptin, adiponectin, and insulin (11).

A nutrition specialist certified as an International Society for the Advancement of Kinanthropometry Level II (ISAK II) Full-profile Technician conducted a nutritional assessment. Initial measurements of anthropometric parameters, including weight, height, BMI, circumferences, and skinfolds, were performed according to the International Standards for Anthropometric Measurements from the Manual published by the International Society for the Advancement of Kinanthropometry. All the saliva, anthropometric, and heart rate measurements were repeated at the end of the intervention period.

In phase IV, interventions were conducted. For the HIIG group, the high-intensity children's games program lasted 16 weeks. It included traditional Colombian games, some similar to HIIT, such as playing tag in the yard, relay races, and children's rounds, as specified in the study by van Biljon et al. (12). Activities in this group were repeated in three sessions per week. The training dynamics were designed based on studies by Reyes-Amigo et al. (13). Games lasted 6 min, followed by a 2-min break, with four games per session. Each session consisted of a 5-min warm-up, 36 min of intervention, and 8 min of stretching. The intensity ranged between 85 and 95% of maximum heart rate (HRmax), continuously monitored using Polar H10^®^ chest straps (Polar Electro Oy, Finland) and the Polar Club^®^ group software.

For the control group, the moderate-intensity physical activity program also lasted 16 weeks. Moderate-intensity activities resembled regular physical education classes, including three stages: a 10-min warm-up consisting of jogging in place for 1 min, jumping jacks for 1 min, and forward and backward sprints for 1 min. The main session involved aerobic exercises for 10 min at 65% of HRmax, with 5-min breaks for a total of 30 min, concluding with 10 min of stretching and cool-down, continuously monitored using Polar H10^®^ chest straps and the Polar Club^®^ group software.

In addition to these interventions, nutritional education sessions were conducted by a nursing professional with both groups of children. A booklet for teachers, parents, and guardians was also provided and explained to the children during nutritional education and healthy lifestyle sessions.

### Statistical analysis

The analysis was performed using the Stata 15^®^ statistical package. Initially, a univariate descriptive analysis was conducted, categorizing variables based on their level of measurement. The Shapiro-Wilk test was applied to assess the normality of quantitative variables, which were described using measures of central tendency (mean) and dispersion (standard deviation). Discrete variables were presented using frequencies and proportions. Subsequently, a bivariate analysis was performed using parametric tests for variables with a normal distribution (Student's *t*-test and paired Student's *t*-test) and non-parametric tests for those without a normal distribution (Wilcoxon signed-rank test and Mann-Whitney U test). Intra-group means were compared before and after the intervention by calculating mean differences using a two-way ANOVA, applying the Šídák test for multiple comparisons. A significance level of P<0.05 was adopted for all statistical tests.

## Results

**Figure 2:**
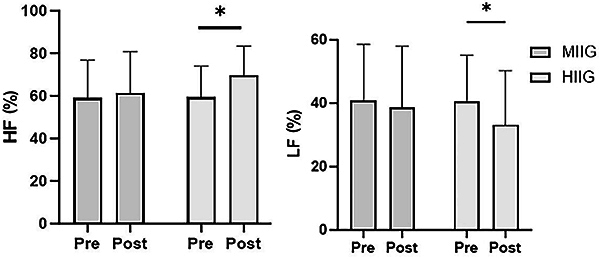
Mean low frequency (LF) and high-frequency (HF) before and after the intervention in the groups of moderate-intensity interval games (MIIG, n=29) and high-intensity interval games (HIIG, n=33). Data are reported as mean and SD. *P<0.05; two-way ANOVA and Šídák's multiple comparisons test.

A total of 67 subjects were included in the study, randomized into two groups: the intervention group (HIIG) and a control group (MIIG). Of these subjects, 62 completed the program and follow-up (30 boys and 32 girls) (Table 1). The different dimensions of HRV were compared both between and within groups before and after the intervention period. No statistically significant differences were found in the MIIG group (Table 2), while the HIIG group showed differences in the frequency domain (Table 3). We observed an increase in HF from 59.3 to 65.8 nu (P=0.04), reflecting greater heart rate variability associated with increased parasympathetic dominance, as well as a decrease in low frequency power (LF) from 40.6 to 34.13 nu (P=0.03), which could be related to reduced sympathetic nervous system dominance, a topic still under debate (Figure 2).

**Table 2:** Heart rate variability of the moderate-intensity intensity game (MIIG) group before and after the intervention.

Parameters	Pre-MIIG	Post-MIIG	%Δ	P
Methods in the time domain				
RR (ms)	741.1 (99.3)	749.1 (103.8)	+7.9 (1.0)	0.51
HR (bpm)	83.1 (11.1)	82.4 (11.2)	-0.68 (0.8)	0.62
SDNN (ms)	69.9 (11.1)	81.9 (11.2)	+12.0 (14.6)	0.44^a^
RMSSD (ms)	78.4 (47.5)	96.0 (83.4)	+17.5 (18.3)	0.62^a^
NN50	149.4 (76.3)	151 (95.5)	+1.5 (1.0)	0.81^a^
pNN50 (%)	38.9 (30.4)	40.0 (29.8)	+1.10 (2.7)	0.93^a^
Methods in the frequency domain				
LF (nu)	40.9 (17.6)	38.5 (19.4)	-2.3 (5.8)	0.41
Hf (nu)	59.0 (17.6)	61.4 (19.4)	+2.3 (3.9)	0.52^a^
Lfnu/Hfn ratio	1.0 (1.4)	0.84 (0.7)	-0.13 (16.0)	0.63^a^
Nonlinear methods				
SD1 (ms)	54.4 (34.0)	67.9 (59.0)	+13.5 (19.8)	0.54^a^
SD2 (ms)	80.9 (30.8)	92.3 (63.8)	+11.4 (12.3)	0.58^a^
ISC (ratio)	1.8 (0.7)	1.7 (0.7)	-0.1 (5.5)	0.52^a^
IVC (ratio)	3.52 (0.4)	3.59 (0.5)	+0.1 (1.94)	0.74^a^

Data are reported as mean and SD (n=29). ^a^Paired sample Student's *t*-test; Wilcoxon signed-rank test. RR: average time interval between successive R-R beats; HR: heart rate; SDNN: standard deviation of R-R intervals; RMSSD: root mean square of successive differences; NN50: number of normal complexes with a difference of more than 50 ms; pNN50: percentage beats with a difference of more than 50 ms; LF: low frequency; HF: high frequency; nu: normalized units; ms: milliseconds; bpm: beats per minute; SD: standard deviation in Poincaré plot; ISC: cardiac sympathetic index; IVC: cardiac vagal index.

**Table 3:** Heart rate variability parameters of the high-intensity interval game (HIIG) group before and after the intervention.

Parameters	Pre-HIIG	Post-HIIG	Δ	P
Methods in the time domain				
RR (ms)	748.9 (82.9)	738.8 (99.5)	-10.1	0.52
HR (bpm)	82.2 (9.0)	83.4 (10.7)	+1.1	0.50^a^
SDNN (ms)	85.0 (69.9)	72.3 (35.0)	-12.7	0.10^a^
RMSSD (ms)	93.5 (51.9)	84.1 (55.1)	-9.44	0.51^a^
NN50 (ms)	161.9 (76.8)	154.1 (88.5)	-7.7	0.71^a^
pNN50 (%)	41.91 (21.7)	40.64 (25.72)	-1.2	0.83^a^
Methods in the frequency domain				
LF (nu)	40.6 (14.6)	34.13 (17.5)	-6.4	0.04*
Hf (nu)	59.3 (14.6)	65.8 (17.5)	+6.48	0.03*
Lfnu/Hfnu (ratio)	0.8 (0.70)	0.65 (0.5)	-0.17	0.20^a^
Nonlinear methods				
SD1 (ms)	66.0 (36.8)	59.5 (39.0)	-6.5	0.51^a^
SD2 (ms)	98.7 (8.7)	84.1 (6.0)	-14.5	0.20^a^
ISC (ratio)	1.62 (.08)	1.7 (.11)	+0.08	0.22
IVC (ratio)	3.7 (0.43)	3.5 (0.4)	-0.1	0.21^a^

Data are reported as mean and SD (n=33). *P<0.05; ^a^Student's *t*-test for paired samples; Wilcoxon signed-rank test. RR: average time interval between successive R-R beats; HR: heart rate; SDNN: standard deviation of R-R intervals; RMSSD: root mean square of successive differences; NN50: number of normal complexes with a difference of more than 50 ms; pNN50: percentage beats with a difference of more than 50 ms; LF: low frequency; HF: high frequency; nu: normalized units; ms: milliseconds; bpm: beats per minute; SD: standard deviation in Poincaré plot; ISC: cardiac sympathetic index; IVC: cardiac vagal index.

Additionally, an 18.6% decrease in the LFnu/HFnu ratio was observed, which, although not statistically significant (P=0.20), may indicate a potential trend toward changes in parasympathetic balance (Table 3). Table 4 compares HRV between groups before and after the intervention. No significant differences were found, but HF values in the HIIG group (65.8 nu) after the intervention were higher than those in the MIIG group (61.4 nu) (P=0.42), which could suggest a tendency toward variations in parasympathetic influence in the high-intensity intervention group.

**Table 4:** Comparison of heart rate variability parameters between the moderate-intensity interval game (MIIG) group and the high-intensity interval game (HIIG) group after the intervention.

Parameters	MIIG (n=29)	HIIG (n=33)	Δ	P
Methods in the time domain				
RR (ms)	749.1 (19.2)	738.8 (17.3)	-10.3	0.69^a^
HR (bpm)	82.4 (1.5)	83.4 (2.0)	+0.9	0.71^a^
SDNN (ms)	81.9 (11.1)	72.3 (6.1)	-9.6	0.84
RMSSD (ms)	96.0 (15.4)	84.1 (9.6)	-11.8	0.93
NN50	151.0 (17.9)	154.1 (15.4)	+3.18	0.92
pNN50 (%)	40.0 (4.9)	40.6 (4.60)	+0.62	0.91
Methods in the frequency domain				
LF (nu)	38.5 (19.4)	34.13 (17.5)	-4.4	0.81
Hf (nu)	61.4 (19.4)	65.8 (17.5)	+3.7	0.42
Lfnu/Hfnu (ratio)	0.8 (0.13)	0.6 (0.09)	-0.74	0.41
Nonlinear methods				
SD1	67.9 (10.9)	59.5 (6.8)	−8.4	0.92
SD2	92.3 (11.8)	84.1 (6.0)	−8.1	0.92
ISC (ratio)	1.7 (0.13)	1.7 (0.11)	-0.01	0.72
IVC (ratio)	3.5 (0.1)	3.5 (0.08)	-0.01	0.90

Data are reported as mean and SD (n=62). ^a^Mann Whitney U test; Student's *t*-test. PromRR: average of the intervals between successive R-R beats; HR: heart rate; SDNN: standard deviation of R-R intervals; RMSSD: root mean square of successive differences; NN50: number of normal complexes with a difference of more than 50 ms; %pNN50: percentage of beats with a difference of more than 50ms; LF: low frequency; HF: high frequency; nu: normalized units; ms: milliseconds; bpm: beats per minute; SD: standard deviation in Poincaré plot; ISC: cardiac sympathetic index; IVC: cardiac vagal index.

**Figure 3:**
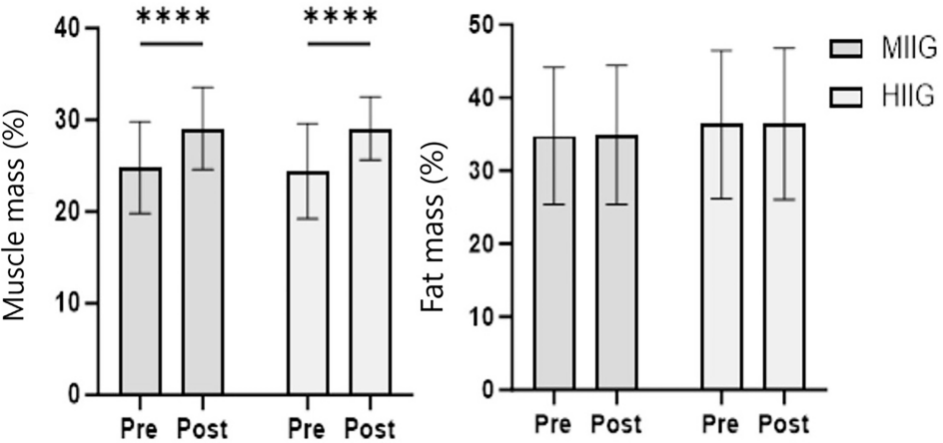
Percentage of muscle mass and fat mass before and after the intervention in the groups of moderate-intensity interval games (MIIG, n=29) and high-intensity interval games (HIIG, n=33). Data are reported as mean and SD. ****P<0.001; Student's *t*-test and Šídák's multiple comparisons test.

The analysis of the effect of high-intensity childhood games on anthropometric measurements showed an increase in height and weight (Table 5), corresponding to the children's growth during the intervention period. A significant increase in muscle mass was observed in both groups (P<0.0001), without an increase in the percentage of body fat (Figure 3). However, the HIIG group showed a statistically significant decrease in the waist-to-hip ratio (P<0.006) compared to the control group.

**Table 5:** Comparison of anthropometric measures before and after the intervention between the moderate-intensity interval game (MIIG) group and the high-intensity interval game (HIIG) group, as well as within each group.

Characteristics	MIIG (n=29)	HIIG (n=33)	Δ	P-value
Height (cm)				
Pre-intervention	133.9 (6.9)	130.2 (6.1)	+3.60	**0.03**
Post-intervention	136.6 (6.7)	133.0 (6.0)	+3.60	**0.02**
Within-group P-value	**0**.**0001**	**0**.**0001**		
Weight (kg)				
Pre-intervention	38.1 (6.3)	36.1 (6.6)	-1.95	0.20
Post-intervention	40.4 (6.5)	38.9 (7.1)	-1.50	0.37
Within-group P-value	**0**.**0001**	**0**.**0001**		
BMI kg/m^2^				
Pre-intervention	21.5 (2.6)	21.2 (2.7)	-0.30	0.62
Post-intervention	21.6 (2.7)	21.8 (2.6)	+0.17	0.74
Within-group P-value	0.521	0.801		
Body fat percentage				
Pre-intervention	34.7 (9.3)	36.3 (10.1)	+1.50	0.53
Post-intervention	34.8 (9.5)	36.4 (10.3)	+1.50	0.54
Within-group P-value	0.61	0.81		
Muscle mass percentage (Poortman method)				
Pre-intervention	24.8 (4.9)	24.4 (5.2)	-0.37	0.91^a^
Post-intervention	29.0 (4.4)	29.0 (3.4)	0.06	0.92
Within-group P-value	**0**.**001**	**0**.**0001**		
Waist-to-hip ratio				
Pre-intervention	0.86 (0.04)	0.88 (0.05)	+0.01	0.27
Post-intervention	0.84 (0.04)	0.85 (0.05)	+0.009	0.44
Within-group P-value	0.071	**0.0006**		

Data are reported as mean and SD. P-values in bold type are statistically significant. Student's *t*-test for equal variances; ^a^Mann-Whitney U test.

**Figure 4:**
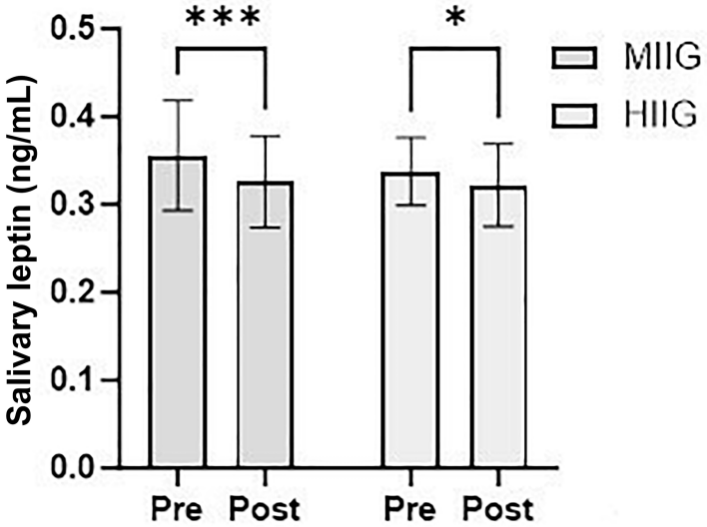
Salivary leptin concentrations before and after the intervention in the groups of moderate-intensity interval games (MIIG, n=29) and high-intensity interval games (HIIG, n=33). Data are reported as mean and SD. ***P<0.001, *P<0.05; Student's *t*-test.

The analysis of salivary concentrations of insulin, TNF-α, IL-6, leptin, and adiponectin before and after the intervention showed significant changes only in leptin concentrations. A statistically significant decrease in salivary leptin concentrations was observed in the MIIG group (P=0.008) (Figure 4). Additionally, although reductions of 39.1% in TNF-α concentrations (P=0.61) and 34.7% in IL-6 were recorded in this group (P=0.08), these changes were not statistically significant. Similarly, a statistically significant decrease in salivary leptin concentrations was found in the HIIG group (P=0.004), with no reductions observed in inflammatory marker concentrations. When comparing salivary biomarker levels between groups, no statistically significant differences were found either before or after the intervention (Table 6).

**Table 6:** Comparative analysis of salivary markers between the moderate-intensity interval game (MIIG) group and the high-intensity interval game (HIIG) group before and after each intervention and intra-group assessments.

Characteristics	MIIG (n=29)	HIIG (n=33)	Δ	P-value
Leptin (ng/mL)				
Pre-test	0.35 (0.06)	0.33 (0.03)	-0.01	0.52
Post-test	0.32 (0.05)	0.32 (0.04)	-0.003	0.80
Intragroup P	0.008*	0.004*		
Insulin (µIU/mL)				
Pre-test	7.3 (4.8)	11.9 (13.7)	+4.61	0.51
Post-test	7.5 (5.8)	9.8 (9.9)	+2.30	0.20
Intragroup P	0.71	0.80		
TNF-α (pg/mL)				
Pre-test	16.6 (29.9)	9.9 (9.72)	-6.60	0.62
Post-test	10.1 (15.2)	10.1 (15.2)	+0.01	0.10
Intragroup P	0.61	0.20		
IL-6 (pg/mL)				
Pre-test	74.7 (176.2)	60.3 (86.5)	-14.30	0.81
Post-test	51.7 (125.7)	66.4 (85.0)	+14.61	0.20
Intragroup P	0.08	0.81		
Adiponectin (ng/mL)				
Pre-test	14.6 (13.3)	16.6 (12.3)	+2.01	0.22
Post-test	11.1 (7.0)	14.8 (13.2)	+3.71	0.61
Intragroup P	0.09	0.11		

Data are reported as mean and SD. *P-values in bold are significantly different. Mann-Whitney U test. TNF-α: tumor necrosis factor alpha; IL-6: interleukin 6.

## Discussion

This study evaluated the effect of HIIG compared to MIIG on cardiac autonomic regulation, obesity biomarkers, and body composition in overweight or obese children. The 16-week intervention included 67 children aged 6 to 9 years, with 62 completing the program.

The main findings showed that the HIIG group experienced significant improvement in HRV, with increased parasympathetic activity (HF, P=0.04) and reduced sympathetic dominance (LF, P=0.03), suggesting a potential reduction in cardiovascular risk. Although no significant differences were found between the groups, a trend toward greater parasympathetic dominance was observed in the HIIG group. In anthropometric terms, both groups increased in height and muscle mass, but only the HIIG group significantly reduced the waist-to-hip ratio (P=0.006), improving body composition. Additionally, a significant decrease in leptin concentrations was recorded in both groups (P=0.008 for MIIG; P=0.004 for HIIG), with no relevant changes in inflammatory markers such as TNF-α or IL-6.

Sex-related differences in HRV dimensions were minimal at baseline. Although girls showed slightly lower values across all dimensions compared to boys before starting the group interventions, these differences were not statistically significant. Leppänen et al. (6) reported similar findings in boys and girls aged 6 to 8, with no differences in SDNN, HF, LF/HF, and RMSSD. Similarly, Speer et al. (14) found no differences between sexes in average R-R interval, resting heart rate, and RMSSD. Likewise, studies by Longin et al. (15) revealed no sex differences in HRV parameters in short-duration recordings.

Regarding the association between HRV and body composition, several studies have demonstrated directly proportional and significant relationships between abdominal circumference and parameters such as LF/HF (16). Some authors propose LF/HF as an indicator of sympathetic-vagal balance (16), as it has proven to be an index reflecting the interaction between the sympathetic and parasympathetic nervous systems. In that study, high waist circumference was found to be associated with a predominance of the sympathetic system over the parasympathetic system in overweight and obese children (17). However, the physiological importance of the LF/HF ratio is not clear in the literature (17). Therefore, the interpretation of LF/HF as an indicator of sympathetic-vagal balance should be approached with caution.

Regarding other variables, the study by Brambilla et al. (18) demonstrated an inverse relationship between HF signal and waist circumference (r=-0.31; P=0.05, and r=-0.38; P<0.01, respectively). The research by Rodríguez-Colón et al. (19) also showed negative associations between these two variables with significant negative regression coefficients (12). These data suggest that central obesity has been inversely related to vagal modulation, which could be explained by increased adipose tissue, leading to the secretion of various adipokines that interact with the hypothalamic-pituitary-adrenal axis (20). This, in turn, increases plasma catecholamine levels and sympathetic tone, with a decrease in the HF band (21).

The LF component reflects not only sympathetic modulation (16) but also parasympathetic modulation, making the interpretation of the sympathetic-vagal balance more complex. However, based on the results, LF appears to be associated primarily with sympathetic activity. Sympathetic hyperactivity related to obesity may function as a compensatory mechanism to increase thermogenesis and reduce weight gain, albeit at the cost of a greater sympathetic burden on the heart, kidneys, and peripheral vasculature (22). It has also been reported that adipose tissue hypertrophy induces resistance of β3 receptors to adrenaline stimulation from the adrenal gland, as well as from the sympathetic nervous system (SNS) (22). All of this leads to increased sympathetic nervous system activity to enhance adipose tissue stimulation, which becomes resistant to this stimulation, so the process ultimately does not occur appropriately, resulting in a decrease in lipolysis (22). However, the increased availability of neurotransmitters created by the sympathetic system and the adrenal gland causes generalized sympathetic stimulation that surpasses parasympathetic regulation, contributing to the pathogenesis of hypertension in obesity (23). Additionally, the known pathway of angiotensinogen production by adipose tissue should be considered (22,23). Therefore, in the frequency domain, it was observed that higher abdominal fat correlates inversely with vagal modulation (HF and LF/HF) and directly with LF (24,25). This finding highlights the utility of this simple measure in assessing autonomic imbalance in children. Although the results may not align with previous studies in many aspects due to methodological differences, this measure could be a valuable tool in the future.

The different methods used for measuring HRV cause confusion and discrepancies in the results. Some studies used 24-h recordings, while others used 5-min recordings. Additionally, discrepancies arise from differences in equipment sensitivity, laboratory environmental conditions (temperature and humidity control), and the emotional state of the subjects being measured. Other disparities could be attributed to variations in pubertal stages across studies, as puberty impacts the function of the autonomic nervous system in obesity (26). Adiposity itself can contribute to differences, given that visceral adipose tissue has a stronger correlation with sympathetic activity than subcutaneous adipose tissue (22). Moreover, BMI calculations are not reliable for quantifying fat percentage or adipose tissue proportion, making it an inadequate indicator for determining whether the adiposity level is adequate (25).

In this study, an HRV analysis was conducted between groups and within each group before and after each intervention period. In the various dimensions of HRV, there were no statistically significant differences for the MIIG group, whereas the HIIG group did exhibit differences in frequency dominance, with an increase in HF (associated with parasympathetic dominance) and a decrease in LF (related, albeit less clearly, to sympathetic dominance). There was also a noteworthy 18.6% decrease in the Lfnu/Hfnu ratio (from 0.8 to 0.6), which, although not statistically significant, suggests some changes in the sympathetic-vagal balance considered beneficial for cardiovascular health in children and adolescents (7). In the study by Farah et al. (27) involving adolescents, significant decreases (P<0.05) were observed in systolic, diastolic, and mean arterial blood pressure in both groups, while beneficial changes in waist circumference, heart rate, and HRV were evident only in the HIIT group (P<0.05). However, Farah et al. (27) faced challenges in participant adherence. In the present study, these issues were addressed by conducting activities in schools and incorporating game-based approaches. Our findings are consistent with the systematic review of previous reports that concluded that physical exercise interventions increase HRV, thereby aligning with more recent discoveries in autonomic modulation among children and adolescents with obesity (28).

There were statistically significant changes in leptin levels in the intragroup analysis, with values ranging from 0.32 to 0.35 ng/mL, significantly lower than the range of 2.0 to 5.6 ng/mL or 12.33 to 15.09 pg/mL reported in the literature (29). Leptin is a crucial hormone that regulates appetite. In obesity, circulating levels of leptin increase, a phenomenon reported as leptin resistance (29). This resistance has been linked to a reduction in leptin transport across the blood-brain barrier and activation of inhibitory negative feedback systems, eventually leading to decreased signaling of the leptin receptor (LepRb) found in neurons of nuclei relevant to metabolic regulation, such as the arcuate nucleus (ARC), ventromedial nucleus (VMH), and dorsomedial nucleus of the hypothalamus (30). Elevated levels of free fatty acids and chronic overnutrition induce lipotoxicity and endoplasmic reticulum (ER) stress, triggering inflammatory responses that may contribute to a less efficient physiological response of leptin in obesity (31). Multiple studies have demonstrated that physical exercise can enhance metabolic regulation and reduce leptin levels due to increased muscle mass, as resistance to leptin appears to affect muscle tissue as well. In this study, participants increased their muscle mass and reduced leptin concentrations, consistent with previous findings (32).

Salivary insulin levels were also measured, revealing values ranging from 7.4 to 11.1 µIU/mL. These findings align with the results reported by Rafiei et al. (33), who compared salivary and plasma insulin values, noting a 1:3 ratio in a group of individuals with appropriate weight and a 1:1.9 ratio in the overweight or obese group, and a positive correlation between blood and saliva values (r=0.66, P=0.01). Fabre et al. (34) demonstrated that saliva insulin concentrations were approximately 10 times lower than serum insulin concentrations with a significant correlation (r=0.92, P<0.001) in a group of 130 boys and 147 girls aged 6 to 14 years. These values closely resemble those found in our study involving minors with obesity, suggesting insulin resistance in our subjects.

In healthy individuals, both pre- and postprandial plasma insulin values typically range from 2.6 to 37.6 µIU/mL (equivalent to pmol/L values of 18.0 to 261.1 in saliva) (35). In our study, most subjects maintained appropriate salivary values; however, it is well-established that individuals with obesity often exhibit elevated insulin levels, further contributing to insulin resistance. Insulin levels remained consistent in both groups before and after the intervention period. Although this study did not reveal significant associations between muscle mass and insulin concentrations, previous research suggests that an increase in muscle mass is linked to improvement in metabolic balance and a potential decrease in postprandial insulin (36).

Another analyte of interest for this research was adiponectin, which is produced in adipocytes as a 28-kDa monomer after various post-translational modifications. It self-assembles into oligomers of different molecular weights - high, medium, and low - with the biologically most active form being the one with high molecular weight. Adiponectin acts through two receptors (AdipoR and the T-cadherin receptor), which are expressed in various organs, tissues, and cell lines, promoting increased insulin sensitivity, enhanced lipolysis, or stimulation of fatty acid oxidation. This molecule is thought to have functions such as reducing plasma levels of triglycerides and other metabolites involved in energy metabolism (37). In this study, the average values for the MIIG group before the intervention were 14.6 ng/mL, and after the intervention, they were 11.1 ng/mL. For the HIIT group, the values before and after the intervention were 16.6 and 14.8 ng/mL, different to studies such as the one by Chávez-Alderete et al. (35), who analyzed salivary analytes in 114 healthy children, finding salivary adiponectin levels ranging from 6.36 to 183.3 ng/mL, indicating a wide range of variation in results.

A decrease in adiponectin levels has generally been associated with obesity (37). However, the values from this research and those of Chávez-Alderete et al. (35) are consistent with other studies, such as Nigro et al. (38). In their analysis of 27 individuals with obesity compared to 27 age- and sex-matched controls, Nigro et al. (38) found a slightly higher expression of adiponectin in obese patients compared to controls (obese patients: 6.1±1.3 ng/mL, controls: 4.8±2.6 ng/mL). Because of the disparity in results, it remains crucial to establish physiological ranges that facilitate clinical decision-making.

To analyze the inflammatory profile of the children, TNF-α levels were measured, yielding values of 16.6 pg/mL (pre-intervention) and 10.1 pg/mL (post-intervention) for the MIIG group, while the HIIG group had values of 9.9 and 10.1 pg/mL, respectively. Nigro et al. (38) also analyzed salivary levels of TNF-α in healthy children and observed values of 12.66 pg/mL, similar to those found in this study. However, as the children studied here were overweight or obese, higher values would be expected due to inflammation. Nigro et al. (38) also evaluated salivary levels of IL-6, finding values of 3.79 pg/mL, contrasting with our results, which had a much higher mean (MIIG: pretest 74.7 pg/mL and post-test 51.7 pg/mL; HIIG: pretest 60.3 pg/mL and post-test 66.4 pg/mL). These results align with findings of low-grade inflammation in individuals with obesity, similar to Sindhu et al. (39), who described higher levels of IL-6 and its receptor in individuals with obesity compared to those with a normal BMI. The high variability observed in IL-6 levels within the MIIG group could be attributed to individual differences in inflammatory responses, as well as factors such as stress, diet, physical activity, and diurnal variations in IL-6 secretion. Additionally, the use of single-point sampling may have contributed to this dispersion. Future research should consider multiple sampling points to reduce variability and include analysis of IL-6 receptor concentrations, as its modulation has been shown to influence the inflammatory profile through receptor blockade (39).

### Limitations

The main limitations of the study were related to the COVID-19 pandemic, which delayed recruitment and led to parental distrust regarding participation in group activities. Although the sample size was similar to that of other studies, the intragroup post-hoc analysis showed a statistical power of 0.62 for HF and 0.61 for LF in the HIIG group, while the between-group statistical power analysis for the RR interval yielded a power of 0.59, indicating that the sample size was moderately adequate. Additionally, the high variability in salivary IL-6 concentrations may have been influenced by factors such as diet, sleep, and physical fitness level.

Future research could increase the sample size and include a control group of children with adequate weight, as well as analyze parental weight and genetic profiles to identify potential associations with childhood obesity. Since consent was only obtained for non-invasive samples, the study was limited to measuring salivary analytes, restricting the ability to correlate them with blood markers, which would have provided more comprehensive data on the utility of salivary measurements in children. Furthermore, determining the HOMA index to assess metabolic dysfunction and considering that salivary density may vary depending on the subjects' hydration status would be beneficial.

## Conclusions

HIIG had a greater impact on HRV than MIIG, improving parasympathetic dominance and sympathetic/parasympathetic balance. Improvements also occurred in body composition and muscle mass, suggesting that implementing game-based strategies of both high and moderate intensity brings anthropometric benefits in this age range. Additionally, a decrease in leptin concentrations was achieved for both the moderate- and high-intensity groups compared to pretest measurements in both groups, indicating an improvement in metabolic balance.
